# miR-133-mediated regulation of the Hedgehog pathway orchestrates embryo myogenesis

**DOI:** 10.1242/dev.159657

**Published:** 2018-06-11

**Authors:** Gi Fay Mok, Estefania Lozano-Velasco, Eirini Maniou, Camille Viaut, Simon Moxon, Grant Wheeler, Andrea Münsterberg

**Affiliations:** 1 School of Biological Sciences, Cell and Developmental Biology, University of East Anglia, Norwich Research Park, Norwich NR4 7TJ, UK; 2 The Earlham Institute, Norwich Research Park, Colney Lane, Norwich NR4 7UH, UK

**Keywords:** miR-133, Chick embryo, Somite myogenesis, Sonic hedgehog signalling, Gli3, Basement membrane

## Abstract

Skeletal myogenesis serves as a paradigm to investigate the molecular mechanisms underlying exquisitely regulated cell fate decisions in developing embryos. The evolutionarily conserved miR-133 family of microRNAs is expressed in the myogenic lineage, but how it acts remains incompletely understood. Here, we performed genome-wide differential transcriptomics of miR-133 knockdown (KD) embryonic somites, the source of vertebrate skeletal muscle. These analyses, performed in chick embryos, revealed extensive downregulation of Sonic hedgehog (Shh) pathway components: patched receptors, Hedgehog interacting protein and the transcriptional activator Gli1. By contrast, *Gli3*, a transcriptional repressor, was de-repressed and confirmed as a direct miR-133 target. Phenotypically, miR-133 KD impaired myotome formation and growth by disrupting proliferation, extracellular matrix deposition and epithelialization. Together, these observations suggest that miR-133-mediated *Gli3* silencing is crucial for embryonic myogenesis. Consistent with this idea, we found that activation of Shh signalling by either purmorphamine, or KD of *Gli3* by antisense morpholino, rescued the miR-133 KD phenotype. Thus, we identify a novel Shh/myogenic regulatory factor/miR-133/Gli3 axis that connects epithelial morphogenesis with myogenic fate specification.

## Abstract (precis)

**Summary:** Here, using chick embryos, we showed that post-transcriptional silencing of the *Gli3* repressor by miR-133 is required to stably establish the myogenic programme in early somites.

## INTRODUCTION

Skeletal muscle is important for mobility and survival. Its development is a highly regulated process, involving developmental signals and their effector pathways, a hierarchy of transcription factors – the myogenic regulatory factors (MRFs), and post-transcriptional regulation by noncoding RNAs (Buckingham and Rigby, 2014; Mok and Sweetman, 2011).

In vertebrate embryos, skeletal muscles of the trunk and limbs are derived from somites, transient paired segments that form in a regular sequence on either side of the neural tube and notochord (Christ and Scaal, 2008). In response to signals, including Wnt, Shh and Notch (Abou-Elhamd et al., 2015; Abu-Elmagd et al., 2010; Borycki et al., 1999; Johnson et al., 1994; Münsterberg et al., 1995; Rios et al., 2011; Sieiro et al., 2016), the initially epithelial somite undergoes morphogenetic changes and differentiates. On the ventral side, cells dissociate to form the sclerotome, whilst the dermomyotome on the dorsal side remains epithelial and contributes myocyte progenitors to the myotome. Myotome formation initiates at the epaxial lip of the dermomyotome, abutting the neural tube (Gros et al., 2004). Interactions with migrating neural crest cells triggers translocation of dermomyotomal lip progenitors into the myotome, where they orientate, elongate and begin to differentiate into myocytes (Rios et al., 2011; Sieiro et al., 2016).

Shh, derived from the notochord and floor plate, activates myogenesis (Münsterberg et al., 1995), and is essential for the activation of the myogenic determination gene, *Myf5*, in epaxial muscle progenitor cells in mice (Borycki et al., 1999; Gustafsson et al., 2002), or in both epaxial and hypaxial domains in avian embryos (Kahane et al., 2013). *Myf5* activation is mediated via Gli activator proteins, Gli1 and Gli2, acting on a Gli-binding site in the mouse epaxial enhancer (Gustafsson et al., 2002; McDermott et al., 2005; Teboul et al., 2002). In the absence of Shh, the Gli3 repressor inhibits *Myf5* transcription (McDermott et al., 2005). Furthermore, in both avian and mouse embryos, Shh signalling is crucial for the transition from proliferating Pax7-positive progenitors to terminally differentiating myocytes (Kahane et al., 2013).

MicroRNAs (miRNAs or miRs) are short noncoding RNAs that bind to target sites located in 3′UTRs of mRNAs. This interaction leads to inhibition of translation, mRNA cleavage and transcript degradation via deadenylation (Bartel, 2009; Béthune et al., 2012). Through their effects on target gene expression, miRNAs regulate developmental timing and provide robustness to cell fate decisions (Ebert and Sharp, 2012; Hornstein and Shomron, 2006). The miR-1, miR-206 and miR-133 families, comprising miR-1-1/2, miR-206 and miR-133a/b, are encoded by three loci in mouse and human, and by four loci in chicken (Sweetman et al., 2008). One member of each family is produced from the same primary transcripts and they play important roles in regulating proliferation, differentiation and cell fate specification in developing muscle (Horak et al., 2016; Mok et al., 2017). In mouse and chicken embryos, miR-1/miR-133a are expressed in skeletal and cardiac muscle. By contrast, miR-206/miR-133b are expressed in myoblasts of somites, limb buds and head muscles, but not in cardiomyocytes (Darnell et al., 2006; Sweetman et al., 2008). In *Xenopus* and zebrafish, the miR-1, miR-206 and miR-133 families are present in skeletal muscle but are not detected in the heart (Ahmed et al., 2015; Mishima et al., 2009). Together with myocyte enhancer factor-2 (MEF2) proteins, the MRFs regulate expression of *miR-1*, *miR-206* and *miR-133* in somites (Liu et al., 2007; Sweetman et al., 2008) and in C2C12 myoblasts (Rao et al., 2006; Rosenberg et al., 2006). In C2C12 myoblasts, the miR-1, miR-206 and miR-133 families regulate the balance between differentiation and proliferation through interactions with multiple targets (Alteri et al., 2013; Chen et al., 2006; Feng et al., 2013; Goljanek-Whysall et al., 2012).

We previously used miRNA knockdown (KD) in chick somites to show that miR-1, miR-206 and miR-133 negatively regulate BRG1/BRM-associated factor 60 (BAF60) variants BAF60A and BAF60B. This facilitates the preferential incorporation of BAF60C into the BAF protein/BRG1 chromatin remodelling complex required for myogenesis (Goljanek-Whysall et al., 2014). In earlier somites, miR-206 facilitates the complete downregulation of Pax3 in the myotome, ensuring timely transition of myogenic progenitor to committed myoblast (Goljanek-Whysall et al., 2011). Studies in mice showed that miR-133a isoforms are essential for the maintenance of skeletal muscle structure and myofibre identity (Liu et al., 2011), and for controlling brown fat differentiation through targeting Prdm16 (Trajkovski et al., 2012); although deletion of the miR-206/133b cluster did not result in skeletal muscle defects (Boettger et al., 2014). In zebrafish, transcriptomic analysis revealed the importance of miR-1 and miR-133 for sarcomeric actin organization (Mishima et al., 2009). However, the functions of miR-133 in early myogenesis remain unclear.

Here, we characterize the mechanisms that underlie the embryonic phenotype resulting from antagomir-mediated miR-133 KD in avian somites. Impaired myogenesis was evident from reduced expression of MRFs, reduced cell proliferation and impaired growth of the dermomyotome and myotome, as well as reduced actin accumulation and disorganized basement membrane (BM) deposition. Differential transcriptomics of miR-133 KD somites identified negative effects on Shh pathway components, suggesting a role for miR-133 in modulating Shh signalling. Expression of the Gli3 transcriptional repressor was de-repressed after miR-133 KD, and luciferase assays confirmed direct regulation via a functional target site in the Gli3 3′UTR that is complementary to the miR-133 seed sequence. Myotome formation and epithelialization, BM deposition and myogenic differentiation were restored in miR-133 KD somites by concomitant activation of Shh signalling using purmorphamine, a synthetic agonist of the smoothened (Smo) receptor (Sinha and Chen, 2006), or by the concomitant morpholino (MO)-mediated knockdown of Gli3 repressor. Our data identify a novel Shh/MRF/miR-133/Gli3 axis and show that stabilization of myogenic differentiation and growth of the myotome require the negative regulation of Gli3 by miR-133.

## RESULTS

### miR-133 is expressed in nascent myoblasts

The spatiotemporal expression of *miR-133* was determined by whole-mount *in situ* hybridization. In Hamburger Hamilton (HH) stage 15 chick embryos (Hamburger and Hamilton, 1951), miR-133 was detected in early stage somites (sIII/IV) and in differentiating somites. Expression was also seen in the neural tube, brain, anterior notochord, mesonephric duct and heart (Fig. S1A). In early somites, which begin to de-epithelialize ventrally, expression was detected adjacent to the neural tube, where the first myoblasts emerge. The relative expression levels of miR-133 increased as somites matured, and in differentiating somites miR-133 was restricted to the myotome (Fig. S1A,B), as previously reported for older-stage embryos (Goljanek-Whysall et al., 2011; Sweetman et al., 2008).

### miR-133 coordinates cell fate acquisition with somite morphogenesis

**Fig. 1.**
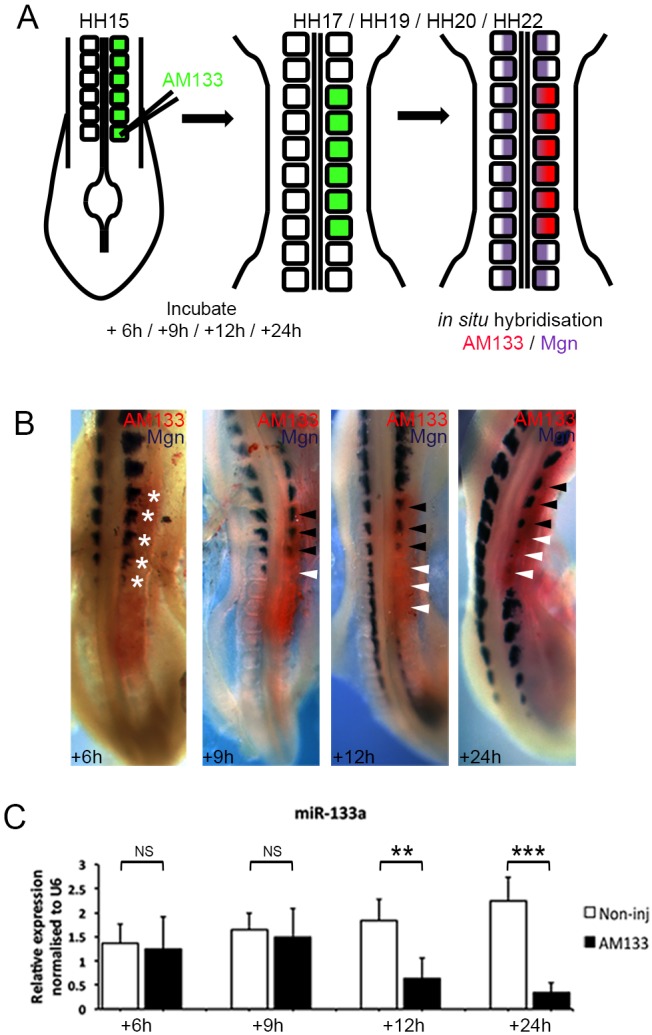
**Inhibition of miR-133 leads to a myogenic phenotype.** (A) Schematic overview of the experimental approach. Posterior somites of HH14/15 embryos were injected with FITC-labelled antagomir-133 (AM133) and the downstream analysis was performed by *in situ* hybridization. (B) Embryos were incubated for 6, 9, 12 and 24 h after AM133 injection as indicated. *In situ* hybridization detects transcripts for myogenin (Mgn, purple) and AM133 is shown in red. After 6 h, there is no change in *Mgn* expression in injected somites (white asterisks, *n*=7/8). After 9 h, the most posterior somites show a loss of (white arrowheads) or reduced (black arrowheads) *Mgn* expression (*n*=12/16). The negative effect on myogenic differentiation becomes more pronounced after 12 h and 24 h (*n*=8/8, *n*=14/14). (C) RT-qPCR for miR-133 of somites, pooled from a minimum of four embryos, injected with AM133 compared with contralateral noninjected somites, harvested after 6, 9, 12 or 24 h of incubation as indicated. ***P<0.001; **P=0.001-0.01; NS, not significant.

Somites are severely affected by antagomir-mediated KD of miR-133, with epithelial morphology lost in the dermomyotome and myotome, and myogenin (*Mgn*) expression either lost completely (80%) or partially (20%), indicating that by 24 h, myogenesis is compromised (Figs 1B and 4B) (Goljanek-Whysall et al., 2014).

To characterize in more detail the underlying cellular and molecular mechanisms, we established that effects resulting from miR-133 inhibition were first detected after 9 h. Epithelial somites of HH14/15 embryos injected with fluorescein isothiocyanate (FITC)-labelled antagomir-133 (AM133) and examined after 6, 9, 12 or 24 h (Fig. 1A) showed partial loss of *Myf5*, *MyoD* (Fig. S2A) and *Mgn* expression 9 h after miR-133 KD (Fig. 1B). No phenotype was detected after 6 h, and after 12 and 24 h, phenotypes were more pronounced (Fig. 1B, Fig. S2B). Control injections of scrambled antagomir (AMscr) had no effect (Fig. S3A-D). Reverse transcription quantitative polymerase chain reaction (RT-qPCR) confirmed significantly reduced abundance of miR-133 in somites after AM133 injection (Fig. 1C), also observed with northern blot analysis (Goljanek-Whysall et al., 2014). Interestingly, AM133 injections of the equivalent, interlimb-level somites at later stages (HH20), when miR-133 is expressed in a more developed myotome, did not lead to myogenic defects (Fig. S2C), suggesting a critical window in younger, less mature somites, in which miR-133 function is essential.

**Fig. 2.**
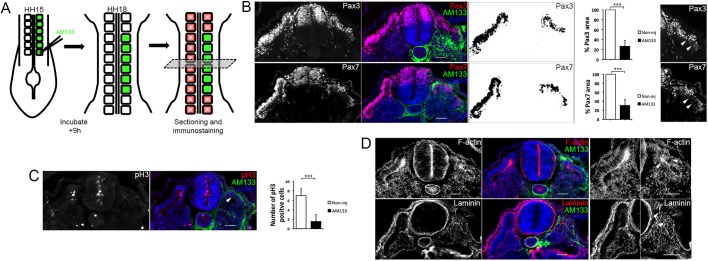
**Dermomyotome growth, epithelial organization and basement membrane deposition are disrupted after inhibition of miR-133.** (A) Schematic overview of the experimental approach. Posterior somites of HH14/15 embryos were injected with FITC-labelled antagomir-133 (AM133) and the downstream analysis was performed by immunostaining after 9 h of incubation. (B) Immunostaining for Pax3, Pax7, AM133 and DAPI as indicated. The areas positive for Pax3 or Pax7 within the somite were quantified using Fiji/ImageJ, and were significantly smaller in AM133-injected somites compared with somites from the noninjected contralateral control side. Higher magnification images of injected somites showed disruption to dermomyotome morphology (white arrowheads). (C) Immunostaining for pH3, AM133 and DAPI as indicated. The number of pH3-positive cells was significantly reduced in AM133-injected somites (white arrowheads) compared with somites from the contralateral side. ***P<0.001. (D) Immunostaining for F-actin, laminin, AM133 and DAPI as indicated. Higher magnification images of noninjected and injected somites stained for F-actin or laminin are shown on the right. White arrowheads indicate disorganized and disrupted staining in the dermomyotome region. Scale bars: 50 μm.

Pax3 and Pax7 immunostaining of cryosections revealed a reduced dermomyotome size, confirmed by pixel measurements using Fiji/ImageJ, in AM133-injected somites compared with somites from the contralateral noninjected side (HH14/15) (Fig. 2A,B). Phosphor histone H3 (pH3) staining showed fewer mitotic cells present in the dermomyotome and myotome, indicating impaired cell proliferation after miR-133 KD (Fig. 2C). Immunostaining for caspase-3 (Cas3) showed no detectable increase in apoptotic cells (Fig. S2D). AMscr injections did not affect dermomyotome proliferation or size (Fig. S3A-C).

Epithelial organization of the dermomyotome, assessed by actin staining, showed a reduced number of apicobasal-orientated dermomyotomal cells. Furthermore, the dermomyotomal lip was poorly defined, had lost its epithelial character and had less actin accumulated than in the same structure in noninjected somites (Fig. 2D). In addition, discontinuous and disorganized laminin staining suggested that BM deposition was impaired, and a BM had not fully formed on the basal side of dermomyotome cells or beneath the myotome (Fig. 2D). The BM surrounding the neural tube remained unaffected, and AMscr injections did not affect epithelial organization of the dermomyotome (Fig. S3D).

### Inhibition of miR-133 affects Shh pathway components

**Fig. 3.**
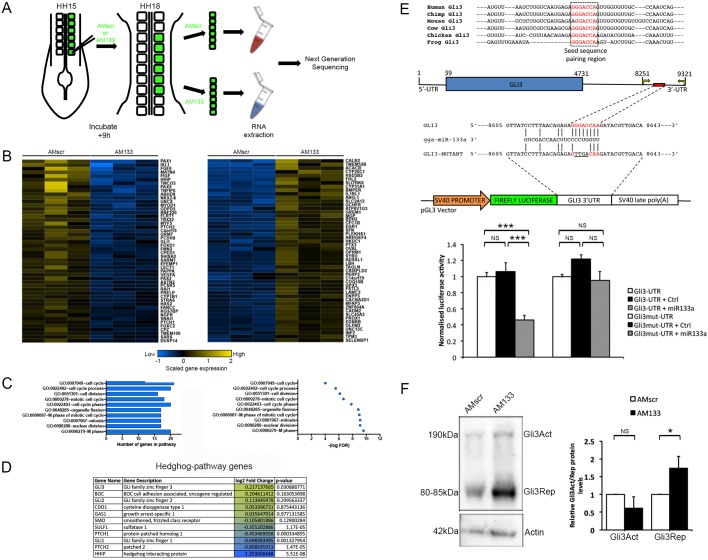
**Differential transcriptomics reveals misregulation of Shh pathway components and identifies Gli3 as a direct miR-133 target.** (A) Schematic overview of the experimental approach. Posterior somites of HH14/15 embryos were injected with FITC-labelled antagomir-133 (AM133) or scrambled-antagomir (AMscr) and harvested after 9 h for RNA isolation and sequencing. (B) Heatmaps of the top 50 genes significantly downregulated or upregulated after miR-133 KD shows clustering of six samples injected with AMscr or with AM133. (C) GO analysis showed that amongst the downregulated DE group, genes associated with cell cycle processes were significantly over-represented. The false discovery rates (FDRs) are shown for these genes. (D) Table showing Hedgehog (Hh) pathway genes that were de-repressed (yellow) and repressed (blue), including the transcriptional regulator *Gli3*, with log2 fold change and *P*-values. (E) Conservation of miR-133 seed sequence pairing region within the *Gli3* 3′UTR of different species. Luciferase assays validate Gli3 as a direct target for miR-133. Schematic of the chick *Gli3* gene with a predicted target site containing an 8-mer seed match (red) present in the 3′UTR. Mutations (underlined) introduced into the predicted target site were designed to disrupt base pairing with the miR-133 seed region. A modified pGL3 vector containing a 1070 bp fragment of the chick *Gli3* 3′UTR downstream of the firefly luciferase reporter gene. Transfection of reporter plasmids into DF1 cells either on their own (white), or with a control miRNA mimic (black), or with miR-133 mimic (grey) confirms negative regulation of the reporter. The response was rescued after mutation of the target site. ***P<0.001; NS, not significant. (F) Western blot of somites injected with AMscr or AM133 shows increased amount of Gli3Rep protein. Quantitative analysis was performed on three biological replicates. *P<0.1; NS, not significant.

The discrete molecular and cellular changes observed after 9 h in the dermomyotome and myotome following AM133 injection culminate in severe impairment of myogenesis by 24 h (Figs 1 and 2, Figs S2 and S3). Genome-wide differential transcriptomics of AM133- or AMscr-injected somites was used to identify the pathways and cellular processes involved (Fig. 3, Fig. S4). This was performed at 9 h to capture the earliest events. Hierarchical clustering confirmed that AM133-injected somites were more similar to each other than to control somites, and identified differentially expressed (DE) genes (Fig. 3B). Gene ontology (GO) analysis showed that the genes involved in processes relating to cell division were significantly decreased (Fig. 3C), consistent with observations that dermomyotome size and number of mitotic cells were reduced after miR-133 KD.

Amongst the top 50 significantly downregulated DE genes were myogenic markers, *Myf5* and *MyoD*, and sclerotome markers, *Pax1* and *Pax9*. Strikingly, several Shh pathway components were also in this group, including patched-1 (*Ptch1*) and patched-2 (*Ptch2*) receptors, Hedgehog interacting protein (*Hhip*) and the transcriptional activator *Gli1* (Fig. 3B,D). Thus, we looked for a transcriptional repressor of Shh pathway genes amongst the genes for which relative expression was increased. A strong candidate was *Gli3*, which was amongst the top 200 de-repressed genes and its expression was significantly increased (*P*=0.03) (Fig. 3D). *Gli3* was therefore a putative direct target gene for miR-133. A miR-133 target site identified in its 3′UTR – conserved in human, chimp, mouse, cow and frog – was validated by luciferase reporter assays. Reporter gene expression was inhibited significantly after transfection of miR-133. Introducing mutations into the 8-mer seed sequence of the target site restored reporter gene expression even in the presence of miR-133 (Fig. 3E), confirming its importance. A control miRNA had no effect. Furthermore, analysis of Gli3 protein levels by western blotting confirmed a relative increase of the short repressor isoform (Gli3Rep) in somites following miR-133 inhibition *in vivo* using AM133 injection compared with control AMscr-injected somites (Fig. 3F).

### Shh pathway activation rescues miR-133 KD

**Fig. 4.**
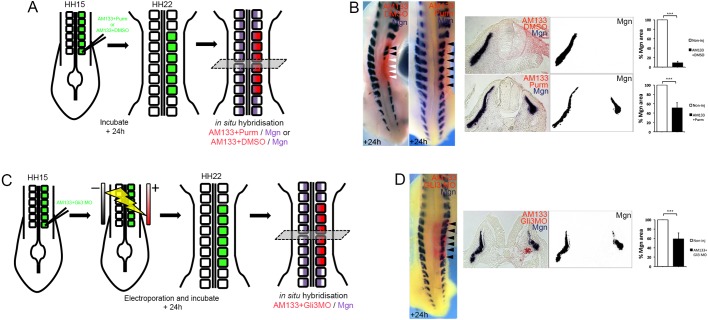
**Pharmacological activation of Shh pathway or Gli3 KD restores myogenesis in the absence of miR-133 function.** (A) Schematic overview of the experimental approach. Posterior somites of HH14/15 embryos were injected with FITC-labelled antagomir-133 (AM133) with purmorphamine (Purm) or FITC-labelled scrambled-antagomir (AMscr) with DMSO as control and the downstream analysis performed by *in situ* hybridization after 24 h incubation. (B) *In situ* hybridization showed that Mgn expression was lost (white arrowheads) after AM133 with DMSO injection, *n*=8/8. Co-injection of AM133 with purmorphamine, a synthetic agonist of the smoothened receptor, rescued myogenesis (black arrowheads). Mgn was expressed and the epithelial nature of the dermomyotome was preserved, but myotome size was reduced, *n*=14/14. Whole mount and sections are shown. (C) Schematic overview of the experimental approach. Posterior somites of HH14/15 embryos were injected with FITC-labelled antagomir-133 (AM133) with *Gli3* MO, electroporated and the downstream analysis performed by *in situ* hybridization after 24 h incubation. (D) Co-transfection of AM133 with *Gli3* MO rescued myogenesis (black arrowheads), although myotome size was reduced (*n*=7/10). Whole mount and sections are shown. Area measurements were obtained using Fiji/ImageJ. ***P<0.001.

The finding that Gli3, which acts predominantly as a transcriptional repressor, is a direct target for miR-133 and de-repressed after miR-133 KD (Fig. 3D,F), led us to test whether pharmacological activation of the Shh pathway can rescue the myogenic phenotype. Knockdown of miR-133 completely inhibited myogenesis after 24 h (Figs 1B and 4B, Fig. S2B). However, co-injection of AM133 and purmorphamine, an activator of Shh signalling (Fig. 4A), restored myogenic differentiation, as shown by expression of Mgn (Fig. 4B). To determine whether Gli3 de-repression was crucial for the phenotype observed, we knocked down *Gli3* expression using MOs and examined whether this could rescue the AM133-induced loss of Mgn. A FITC-labelled *Gli3*-translation-blocking MO was electroporated concomitant with AM133 injection (Fig. 4C,D). Western blots of transfected somites showed that Gli3Rep protein was reduced by *Gli3* MO compared with control MO (Fig. S3F). A faint band representing the full-length *Gli3* activator (Gli3Act) was present in both samples, consistent with the finding that Gli3Act becomes rapidly degraded (Wen et al., 2010). *In situ* hybridization showed that *Gli3* MO restored myogenesis in AM133-treated somites (Fig. 4D), suggesting that miR-133 mediates its effects via negative regulation of Gli3.

**Fig. 5.**
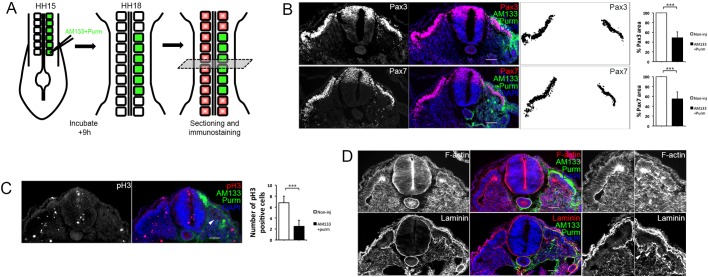
**Shh pathway activation in antagomir-133 injected somites restores dermomyotome morphology, epithelial organization and basement membrane deposition but not proliferation.** (A) Schematic overview of the experimental approach. Posterior somites of HH14/15 embryos were injected with FITC-labelled antagomir-133 (AM133) with purmorphamine (Purm), and the downstream analysis was performed by immunostaining after 9 h incubation. (B) Immunostaining for Pax3 or Pax7, AM133 and DAPI, and area measurements after AM133 with purmorphamine injections. (C) Immunostaining for pH3, AM133 and DAPI, and counting of positive cells after AM133 plus purmorphamine injections. The number of pH3-positive cells was reduced (white arrowhead) in AM133 plus purmorphamine-injected somites compared with somites from the contralateral side. (D) Epithelial organization and basement membrane deposition were improved after co-injection of purmorphamine with AM133. Immunostaining for F-actin, laminin, AM133 and DAPI as indicated. Higher magnification images of noninjected and injected somites are shown on the right, with the dermomyotome, dorsomedial lip and more continuous BM staining beneath the myotome indicated by white arrowheads. Scale bars: 50 μm. A minimum of ten sections from three embryos were analysed for each experiment. ***P<0.001.

Somite organization was improved after treatment with purmorphamine or the concomitant transfection of *Gli3* MO together with AM133; however, the somites were smaller (Fig. 4B,D). Thus, we examined the rescue phenotype in more detail, after 9 h when Mgn expression appeared normal (Fig. S5). Pax3 and Pax7 immunostaining showed that although the dermomyotome was smaller, its epithelial nature was preserved in somites injected with AM133 and purmorphamine (Fig. 5A-D). Fewer mitotic cells were detected by pH3 staining, compared with contralateral somites (Fig. 5C), and this was similar to observations in somites injected with AM133 alone (Fig. 2C). Actin and laminin staining showed that epithelial character and BM deposition were restored around the dermomyotome and myotome (Fig. 5D).

**Fig. 6.**
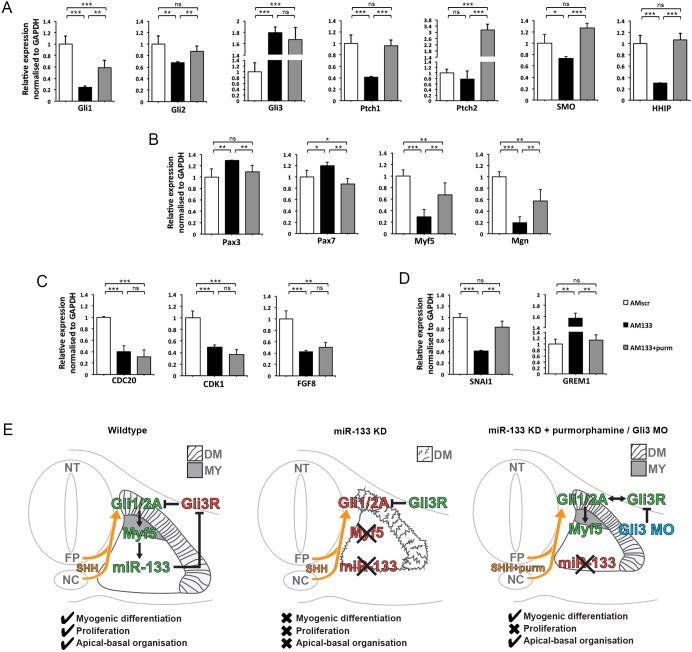
**Purmorphamine restores expression of Shh pathway and myogenic genes; however, *Gli3* and cell cycle genes remain de-regulated in the absence of miR-133 function.** (A-D) RT-qPCR for Shh pathway components (A), myogenic genes (B), cell cycle genes (C) and a regulator of EMT and BMP signalling (D), as indicated. White columns represent somites injected with scrambled antagomir (AMscr), black columns represent somites injected with antagomir-133 (AM133), grey columns represent somites injected with AM133 with purmorphamine. (E) Summary of the regulatory interactions identified in this study. DM, dermomyotome; FP, floor plate; MY, myotome; NC, notochord; NT, neural tube.

Finally, AM133 co-injection with purmorphamine restored expression levels of DE hedgehog pathway genes (Fig. 6A). Somites injected with AMscr, or with AM133 alone, or with AM133 and purmorphamine, were examined using RT-qPCR. Expression of *Gli1*, *Gli2*, *Ptch1*, *Smo* and *Hhip* was decreased in somites injected with AM133, whereas expression of *Gli3* increased, consistent with the differential transcriptomics data. Purmorphamine co-injection with AM133 led to recovery of Shh pathway components compared with somites injected with AM133 alone. The exception was *Gli3* expression, which remained de-repressed. This was not unexpected given that miR-133 function was still inhibited by antagomir-133 (Fig. 6A). The relative expression of *Pax3* and *Pax7* increased, whilst that of *Myf5* and *Mgn* decreased, after antagomir-133 injection. *Myf5* was also significantly decreased after miR-133 KD in the differential transcriptomics data (Fig. 3B). Co-injection of purmorphamine with AM133 restored expression of pre-myogenic markers, *Pax3* and *Pax7*, to levels comparable to those of control somites, and *Myf5* and *Mgn* levels were also rescued (Fig. 6B). In addition, expression of *Snail1*, a gene associated with epithelial mesenchymal transition (EMT), or gremlin 1 (*Grem1*), a BMP antagonist was rescued by Shh pathway activation (Fig. 6D). However, interestingly, genes associated with proliferation, *Cdc20*, *Cdk1* and *Fgf8*, remained repressed, consistent with fewer pH3-positive cells and smaller somites in the presence of AM133, irrespective of the presence of purmorphamine. This suggests that myogenic differentiation and epithelialization are uncoupled from proliferation and that there is differential sensitivity of these processes for GliAct/GliRep balance. Furthermore, there might be additional functions of miR-133, independent of Gli3 targeting and Shh pathway regulation.

## DISCUSSION

Despite recent progress, we still do not have a complete understanding of how myogenic progenitor cells, once specified, can stably execute their differentiation programme. miRNAs are involved in the fine tuning of developmental processes, and we use the accessibility of chicken embryos to investigate the function of the miR-133 family *in vivo*, during embryonic myogenesis using antagomir-mediated inhibition, differential transcriptomics and rescue experiments.

Embryonic loss of function of miRNAs is often difficult to investigate owing to functional redundancy of almost identical mature miRNAs produced from multiple genetic loci, thus making reverse genetic approaches in mice challenging. Observations from mice with genetic deletion of miR-133a-1 and miR-133a-2 family members suggest a role for adult skeletal muscle homeostasis (Liu et al., 2011). By contrast, removal of the miR-206/133b cluster did not reveal essential functions in skeletal muscle differentiation or regeneration (Boettger et al., 2014). The concomitant knockout (KO) of the miR-1-1/133a-2 and miR-1-2/133a-1 clusters, which are expressed in cardiac and skeletal muscle, led to an early cardiac defect (Wystub et al., 2013). However, an embryonic skeletal muscle phenotype was not reported in any of these lines, most likely due to expression from unaffected loci. Chick embryos offer the opportunity to perform conditional miRNA KD experiments using antagomirs that simultaneously inhibit the mature form of all miR-133 family members produced (Goljanek-Whysall et al., 2014). This has uncovered a crucial function of miR-133 in the modulation of Shh signalling through direct targeting of *Gli3*, a transcriptional repressor of the pathway and of myogenesis (McDermott et al., 2005; Wen et al., 2010).

Shh signals, derived from the notochord and floorplate, activate myogenesis in explants of presegmented mesoderm and act on myogenic progenitors in the dermomyotome and dermomyotome lip (Borycki et al., 1999, 1998; Münsterberg et al., 1995). This induces expression of *Myf5* via Gli activator proteins. Myf5 activates expression of miR-133 via upstream E-boxes (Rao et al., 2006; Rosenberg et al., 2006; Sweetman et al., 2008), and we show here that miR-133 directly targets *Gli3* via a conserved site in the 3′UTR (Fig. 3E). This site is conserved and was shown to be functional in human Sertoli cells (Yao et al., 2016). We suggest a model in which *Gli3* silencing by miR-133 maintains the finely tuned balance of Gli activator and repressor forms during myogenesis (Fig. 3F). Thus, post-transcriptional silencing of *Gli3* in nascent myoblasts promotes the stable activation of the skeletal muscle differentiation programme in response to the Gli activators Gli1 and Gli2 (Fig. 6E). This model is consistent with the finding that concomitant *Gli3* knockdown using a MO restored myogenic differentiation after miR-133 KD (Fig. 4D). In addition, miR-133 expression, which is initiated by Myf5 in early myoblasts, is mutually exclusive with Gli3 (Fig. S1) (Berti et al., 2015; Mok et al., 2015; Sweetman et al., 2008). In early somites, *Gli3* transcripts are excluded from Myf5/miR-133-expressing myoblasts and restricted to the dermomyotome, including the dermomyotome lip (Fig. S1C). In differentiating somites, in which the myogenic programme is stably established, miR-133 and Gli3 remain expressed in a mutually exclusive fashion, in the myotome or dermomyotome, respectively (Goljanek-Whysall et al., 2014; Kahane et al., 2013). In later-stage somites, AM133 injection had no effect on myogenin expression (Fig. S2C), suggesting that miR-133 function is essential specifically during early myogenesis. In addition, the lack of a detectable phenotype at later stages shows that there are no nonspecific, off-target or toxic effects.

De-repression of *Gli3* transcript and protein following miR-133 KD led to inhibition of the Shh pathway (Figs 3B,D,F and 6A), including the downregulation of Gli1 activator, which is important for *Myf5* activation (Gustafsson et al., 2002; McDermott et al., 2005). Our results imply direct and indirect consequences resulting from disruption of the Gli1/2 and Gli3 balance in early somites. It is likely that early myoblasts are directly affected, with miR-133 KD leading to loss of stable myogenesis, after initial expression of MRFs (Fig. 1B). The finding that concomitant *Gli3* KD, using electroporation of a *Gli3* MO (Fig. 4D, Fig. S3F), rescued myogenic gene expression suggests that miR-133 mediated post-transcriptional regulation of *Gli3* is crucial to stably establish the myogenic programme. However, myogenic differentiation also involves extracellular matrix production and thus dermomyotome epithelial organization could be affected indirectly.

Shh signal response genes expressed in the myotome include *Ptch1* and *Ptch2* (Pearse et al., 2001), *Gli1*, *Hhip* (Kahane et al., 2013), which attenuates signalling (Chuang and McMahon, 1999; Ingham and McMahon, 2001), *Fgf8* (Smith et al., 2005) and the myogenic determination gene, *Myf5* (Borycki et al., 1998; McDermott et al., 2005). Differential transcriptomics and RT-qPCR data showed that these genes are negatively affected by miR-133 KD in developing somites (Figs 3B,D and 6A-C). Thus, they are secondary targets of miR-133, but might be directly regulated by *Gli3* repressor (Fig. 3F). Using motif searches we found potential Gli binding sites within 2 kb upstream of transcription start sites in chicken *Ptch1*, *Ptch2*, *Myf5* and *Fgf8* genes, consistent with *Ptch1* and *Myf5* being direct targets for Gli proteins (Cohen et al., 2015; Gustafsson et al., 2002).

Other genes downregulated after miR-133 inhibition, such as cell cycle associated genes, *Cdk1* and *Cdc20*, or sclerotome genes, *Pax1* and *Pax9* (Fig. 3B), are likely to be indirectly affected through feedback mechanisms and/or noncell-autonomous mechanisms. For example, the significant downregulation of *Fgf8* could explain effects on cell proliferation and growth as well as on sclerotome differentiation (Figs 3B and 6C). In addition, BMP signals cooperate with Shh to activate somitic chondrogenesis (Murtaugh et al., 1999; Zeng et al., 2002). BMP signalling is likely to be inhibited after miR-133 KD, because *Grem1*, a BMP antagonist, is amongst the top 50 de-repressed genes (Fig. 3B). This provides a possible explanation for negative effects on Pax1 and Pax9 expression. Interestingly, in developing limb buds, Gli3 specifies digit identities by promoting cell cycle exit and BMP-dependent chondrogenic differentiation via controlling *Grem1* expression (Lopez-Rios et al., 2012).

The role of Gli2 is less clear at present. Although we cannot exclude that Gli2 also contributes to the negative regulation of Shh pathway components, we think that this is less likely. Differential transcriptomics showed that *Gli2* was slightly de-repressed; however, this change was not significant (Fig. 3D) and was not confirmed by RT-qPCR (Fig. 6A), and the *Gli2* 3′UTR has no predicted miR-133 target site. Expression patterns of Gli transcription factors in chick somites are more consistent with the idea that *Gli3* is the main repressor of the myogenic programme: Gli1 and Gli2 are expressed in both dermomyotome and myotome; however, Gli3 is excluded from the myotome (Borycki et al., 1998; Kahane et al., 2013).

The effect of miR-133 KD on somite differentiation is dramatic. Expression of myogenic differentiation genes and epithelial organization of the dermomyotome and myotome are severely affected (Figs 1 and 2, Fig. S2A,B). It has been shown that Shh is important for laminin alpha 1 synthesis in the myotome (Anderson et al., 2009), thus suggesting miR-133 KD, and the resulting *Gli3* de-repression might lead to disrupted BM assembly, owing to effects on laminin activation. Co-injection with purmorphamine restored BM deposition, indicating that rescue of Gli1, and to some extent Gli2, expression restores the balance of Gli proteins sufficiently to allow laminin synthesis, even in the presence of elevated Gli3 levels (Figs 4E and 5D).

Shh is also required to maintain the epithelial character of the dermomyotome (Kahane et al., 2013), which was disrupted upon miR-133 KD (Fig. 2C) and rescued by purmorphamine co-injection with AM133 (Fig. 5D). Purmorphamine-mediated rescue confirms that stabilization of the myogenic differentiation programme is intimately linked with cellular organization, and both depend on Shh pathway activity (Fig. 6E). This is in line with the close integration of epithelial morphology and cell fate determination mediated by Notch, GSK3β and Snail1 during the initiation of myogenesis (Sieiro et al., 2016). On the other hand, myogenesis and epithelialization were uncoupled from proliferation. Proliferation was not rescued owing to the continued de-repression of *Gli3*, which might affect expression of mitotic signals, such as *Fgf8*, and cell cycle regulators, *Cdc20* and *Cdk1* (Fig. 6A,C). Similar observations have previously been made in the limb (Lopez-Rios et al., 2012) and the neural tube (Cayuso et al., 2006; Ulloa et al., 2007). Finally, it has been reported that Shh causes premature myogenic differentiation (Borycki et al., 1999; Kahane et al., 2001, 2013), which could also explain why proliferation was not rescued.

Together, our data uncover a novel Shh/MRF/miR-133/Gli3 axis by which miR-133 and its modulation of the Shh signalling pathway via the direct targeting of Gli3 enable the coordination of epithelial morphology with stabilization of the cellular differentiation programme during early myogenesis.

## MATERIALS AND METHODS

### Somite injections

Fertilized eggs (Henry Stewart) were incubated until the desired stage of development (Hamburger and Hamilton, 1951). Antagomir-133 (AM133) and a scrambled sequence (AMscr), with final concentration of 1 µM, were designed as previously described (Goljanek-Whysall et al., 2011). The posterior six somites of HH14/15 embryos, or the equivalent interlimb-level somites of HH20 embryos, were injected. Embryos were harvested and processed for *in situ* hybridization or immunohistochemistry, or injected somites were dissected and processed for RNA or protein extraction. Purmorphamine (Sigma-Aldrich) was dissolved in dimethyl sulfoxide (DMSO) (2 μM) (Dessaud et al., 2007) and co-injected with antagomir. Gli3 antisense MO was 3′ FITC-labelled (Gene Tools) (Table S1). Gli3 MO was co-injected with AM133 into the posterior six somites of HH14/15 embryos, followed by electroporation using six 10-ms pulses of 60 V.

### *In situ* hybridization, immunohistochemistry and image analysis

Whole-mount *in situ* hybridization using digoxigenin-labelled LNA oligo probe for miR-133a (Exiqon) or antisense RNA probes for *Pax3*, *Myf5*, *MyoD*, *Mgn*, *Gli3* (a gift from Matt Towers, University of Sheffield, Sheffield, UK) was carried out as described previously (Goljanek-Whysall et al., 2011). Antagomirs were detected using anti-FITC antibody coupled to alkaline phosphatase (Roche) as previously described (Goljanek-Whysall et al., 2011). Cryosections (15 μm) of 4% paraformaldehyde-fixed OCT embedded embryos were immunostained. Primary antibodies used were anti-Pax3 (1:200), anti-Pax7 (1:200), anti-laminin (1:100), from the Developmental Studies Hybridoma Bank, and anti-rabbit pH3 (1 mg/ml, Merck). Phalloidin (Invitrogen) was used at 1:100 to stain actin. Secondary antibodies were Alexa Fluor 647-conjugated anti-rabbit or anti-mouse (Invitrogen), used at 1 mg/ml in 5% bovine serum albumin/5% goat serum/PBS. DAPI (Sigma-Aldrich) was used at 0.1 mg/ml in PBS. Sections were visualized on an Axioscope with Axiovision software (Zeiss). Images were imported into Fiji/ImageJ, and areas of staining were calculated from binary images by calculating pixel numbers from injected and noninjected sides, when appropriate neural tube staining was removed. A minimum of 10 sections from three embryos were analysed for each experiment. Statistical analysis used GraphPad Prism (version 6) software. Mann–Whitney nonparametric two-tail testing was applied to determine *P*-values.

### RNA extraction and RT-qPCR

RNA and miRNA isolation from somites was performed using RNeasy (Qiagen) and miRCURY RNA kits (Exiqon) according to the manufacturer's protocols. cDNA was synthesized from 600 ng RNA using a Maxima First Strand cDNA synthesis kit (Thermo Fisher Scientific). For miRNAs, cDNA was synthesized from 10 ng using Universal cDNA synthesis kit II (Exiqon). RT-qPCR was performed on a 7500 Fast Real Time PCR machine (Applied Biosystems) using SYBR Green PCR Master Mix (Thermo Fisher Scientific) according to the manufacturer's instructions. Primers for the miR-133a sequence 5′-UUGGUCCCCUUCAACCAGCUGU-3′, were designed by Exiqon. Other primers (Sigma-Aldrich) (Table S1) were designed with Primer3 software (http://biotools.umassmed.edu/bioapps/primer3_www.cgi). RT-qPCR was normalized to *Gapdh* for mRNA, or *Rnu6* for miRNA, based on Exiqon protocols. Three independent experiments each with three replicate samples were performed for each RT-qPCR. The ΔΔCT (Livak and Schmittgen, 2001) method was used to analyse gene expression levels. Statistical analysis was performed as previously described.

### RNA sequencing

Sequencing libraries were built according to Illumina Standard Protocols (Earlham Institute). Each sample contained pooled, injected somites from ten embryos. Sequencing was performed on one lane of a flow cell on an Illumina HiSeq2500 platform. cDNA was end-paired, A-tailed and adapter-ligated before amplification and size selection. Library QC used a gel and a bioanalyser. Transcript abundances for each sample were estimated with Kallisto (Bray et al., 2016) using the *Gallus gallus* reference cDNA set (Galgal5) downloaded from Ensembl (Yates et al., 2016). Differential expression between antagomir-133 (AM133)- and scrambled (AMscr)-injected samples was calculated using DESeq (Anders and Huber, 2010), with an adjusted *P*-value significance threshold of 0.05. The data have been uploaded to the NCBI SRA, under accession number PRJNA384007. GO term analysis was performed using the DAVID Bioinformatics Resources 6.8, available at https://david.ncifcrf.gov/. Statistical analysis was performed using false discovery rate (FDR).

### DNA constructs, transfections and luciferase assay

Sensor constructs contained a chick *Gli3* 3′UTR fragment in a modified pGL3 vector (Promega); for primers see Table S1. Mutant construct replaced the miR-133a seed site GGGACCA with the sequence GTTGACAA. Chick dermal fibroblast (DF1) cells were transfected in 96-well plates with 200 ng luciferase reporter plasmid with miR-133 or control (50 nM, Sigma-Aldrich) using Lipofectamine 2000 (Invitrogen). A Renilla luciferase plasmid was included to normalize for transfection efficiency and transfections used triplicate samples. The miRNA mimics were identical to mature miRNA; sequences are listed in Table S1. Firefly and Renilla luciferase activity was measured after 24 h using a multilabel counter (Promega GloMax), and relative activity was calculated for each sample.

### Primary cell culture and western blotting

Somites of wild-type embryos were dissected and cultured in Dulbecco's modified Eagle medium, 10% fetal bovine serum, 1% penicillin/streptomycin for 4 h before being transfected with Gli3 MO (1 mM) or control MO (1 mM) using Endoporter PEG (Gene Tools) and protein extracted after 48 h. Somites from AM133- or AMscr-injected embryos were dissected for protein extraction. Protein lysate (31.5 μg) was run on pre-cast 4-15% polyacrylamide gels (Bio-Rad) and blotted onto polyvinylidene fluoride membrane (Bio-Rad). Primary antibody against Gli3 (1:200, 6F5 Gli3N, Genentech, Wen et al., 2010) was applied at 4°C overnight before secondary polyclonal goat anti-mouse HRP (1:1000, P0447, DAKO) was applied for 1 h at room temperature. The blots were treated with an ECL substrate kit (GE Healthcare) and imaged. Primary antibody against actin (1:1000, ab3280, Abcam) was applied at 4°C overnight; secondary polyclonal goat anti-mouse HRP was applied for 1 h at room temperature. The blots were treated with an ECL substrate kit and imaged. Quantification of blots was performed using ImageJ.

## Supplementary Material

Supplementary information

## References

[DEV159657C1] Abou-Elhamd A., Alrefaei A. F., Mok G. F., Garcia-Morales C., Abu-Elmagd M., Wheeler G. N. and Munsterberg A. E. (2015). Klhl31 attenuates beta-catenin dependent Wnt signaling and regulates embryo myogenesis. *Dev. Biol.* 402, 61-71. 10.1016/j.ydbio.2015.02.024 25796573

[DEV159657C2] Abu-Elmagd M., Robson L., Sweetman D., Hadley J., Francis-West P. and Munsterberg A. (2010). Wnt/Lef1 signaling acts via Pitx2 to regulate somite myogenesis. *Dev. Biol.* 337, 211-219. 10.1016/j.ydbio.2009.10.023 19850024

[DEV159657C3] Ahmed A., Ward N. J., Moxon S., Lopez-Gomollon S., Viaut C., Tomlinson M. L., Patrushev I., Gilchrist M. J., Dalmay T., Dotlic D. et al. (2015). A database of microRNA expression patterns in Xenopus laevis. *PLoS ONE* 10, e0138313 10.1371/journal.pone.0138313 26506012 PMC4624429

[DEV159657C4] Alteri A., De Vito F., Messina G., Pompili M., Calconi A., Visca P., Mottolese M., Presutti C. and Grossi M. (2013). Cyclin D1 is a major target of miR-206 in cell differentiation and transformation. *Cell Cycle* 12, 3781-3790. 10.4161/cc.26674 24107628 PMC3905070

[DEV159657C5] Anders S. and Huber W. (2010). Differential expression analysis for sequence count data. *Genome Biol.* 11, R106 10.1186/gb-2010-11-10-r106 20979621 PMC3218662

[DEV159657C6] Anderson C., Thorsteinsdottir S. and Borycki A.-G. (2009). Sonic hedgehog-dependent synthesis of laminin alpha1 controls basement membrane assembly in the myotome. *Development* 136, 3495-3504. 10.1242/dev.036087 19783738 PMC2752398

[DEV159657C7] Bartel D. P. (2009). MicroRNAs: target recognition and regulatory functions. *Cell* 136, 215-233. 10.1016/j.cell.2009.01.002 19167326 PMC3794896

[DEV159657C8] Berti F., Nogueira J. M., Wöhrle S., Sobreira D. R., Hawrot K. and Dietrich S. (2015). Time course and side-by-side analysis of mesodermal, pre-myogenic, myogenic and differentiated cell markers in the chicken model for skeletal muscle formation. *J. Anat.* 227, 361-382. 10.1111/joa.12353 26278933 PMC4560570

[DEV159657C9] Béthune J., Artus-Revel C. G. and Filipowicz W. (2012). Kinetic analysis reveals successive steps leading to miRNA-mediated silencing in mammalian cells. *EMBO Rep.* 13, 716-723. 10.1038/embor.2012.82 22677978 PMC3410385

[DEV159657C10] Boettger T., Wüst S., Nolte H. and Braun T. (2014). The miR-206/133b cluster is dispensable for development, survival and regeneration of skeletal muscle. *Skelet Muscle* 4, 23 10.1186/s13395-014-0023-5 25530839 PMC4272821

[DEV159657C11] Borycki A. G., Mendham L. and Emerson C. P. Jr. (1998). Control of somite patterning by Sonic hedgehog and its downstream signal response genes. *Development* 125, 777-790. 9435297 10.1242/dev.125.4.777

[DEV159657C12] Borycki A. G., Brunk B., Tajbakhsh S., Buckingham M., Chiang C. and Emerson C. P. Jr. (1999). Sonic hedgehog controls epaxial muscle determination through Myf5 activation. *Development* 126, 4053-4063. 10457014 10.1242/dev.126.18.4053

[DEV159657C13] Bray N. L., Pimentel H., Melsted P. and Pachter L. (2016). Near-optimal probabilistic RNA-seq quantification. *Nat. Biotechnol.* 34, 525-527. 10.1038/nbt.3519 27043002

[DEV159657C14] Buckingham M. and Rigby P. W. J. (2014). Gene regulatory networks and transcriptional mechanisms that control myogenesis. *Dev. Cell* 28, 225-238. 10.1016/j.devcel.2013.12.020 24525185

[DEV159657C15] Cayuso J., Ulloa F., Cox B., Briscoe J. and Marti E. (2006). The Sonic hedgehog pathway independently controls the patterning, proliferation and survival of neuroepithelial cells by regulating Gli activity. *Development* 133, 517-528. 10.1242/dev.02228 16410413

[DEV159657C16] Chen J.-F., Mandel E. M., Thomson J. M., Wu Q., Callis T. E., Hammond S. M., Conlon F. L. and Wang D.-Z. (2006). The role of microRNA-1 and microRNA-133 in skeletal muscle proliferation and differentiation. *Nat. Genet.* 38, 228-233. 10.1038/ng1725 16380711 PMC2538576

[DEV159657C17] Christ B. and Scaal M. (2008). Formation and differentiation of avian somite derivatives. *Adv. Exp. Med. Biol.* 638, 1-41. 21038768 10.1007/978-0-387-09606-3_1

[DEV159657C18] Chuang P.-T. and McMahon A. P. (1999). Vertebrate Hedgehog signalling modulated by induction of a Hedgehog-binding protein. *Nature* 397, 617-621. 10.1038/17611 10050855

[DEV159657C19] Cohen M., Kicheva A., Ribeiro A., Blassberg R., Page K. M., Barnes C. P. and Briscoe J. (2015). Ptch1 and Gli regulate Shh signalling dynamics via multiple mechanisms. *Nat. Commun.* 6, 6709 10.1038/ncomms7709 25833741 PMC4396374

[DEV159657C20] Darnell D. K., Kaur S., Stanislaw S., Konieczka J. H., Yatskievych T. A. and Antin P. B. (2006). MicroRNA expression during chick embryo development. *Dev. Dyn.* 235, 3156-3165. 10.1002/dvdy.20956 17013880

[DEV159657C21] Dessaud E., Yang L. L., Hill K., Cox B., Ulloa F., Ribeiro A., Mynett A., Novitch B. G. and Briscoe J. (2007). Interpretation of the sonic hedgehog morphogen gradient by a temporal adaptation mechanism. *Nature* 450, 717-720. 10.1038/nature06347 18046410

[DEV159657C22] Ebert M. S. and Sharp P. A. (2012). Roles for microRNAs in conferring robustness to biological processes. *Cell* 149, 515-524. 10.1016/j.cell.2012.04.005 22541426 PMC3351105

[DEV159657C23] Feng Y., Niu L.-L., Wei W., Zhang W. Y., Li X. Y., Cao J. H. and Zhao S. H. (2013). A feedback circuit between miR-133 and the ERK1/2 pathway involving an exquisite mechanism for regulating myoblast proliferation and differentiation. *Cell Death Dis.* 4, e934 10.1038/cddis.2013.462 24287695 PMC3847338

[DEV159657C24] Goljanek-Whysall K., Sweetman D., Abu-Elmagd M., Chapnik E., Dalmay T., Hornstein E. and Münsterberg A. (2011). MicroRNA regulation of the paired-box transcription factor Pax3 confers robustness to developmental timing of myogenesis. *Proc. Natl. Acad. Sci. USA* 108, 11936-11941. 10.1073/pnas.1105362108 21730146 PMC3141954

[DEV159657C25] Goljanek-Whysall K., Pais H., Rathjen T., Sweetman D., Dalmay T. and Münsterberg A. (2012). Regulation of multiple target genes by miR-1 and miR-206 is pivotal for C2C12 myoblast differentiation. *J. Cell Sci.* 125, 3590-3600. 10.1242/jcs.101758 22595520

[DEV159657C26] Goljanek-Whysall K., Mok G. F., Fahad Alrefaei A., Kennerley N., Wheeler G. N. and Munsterberg A. (2014). myomiR-dependent switching of BAF60 variant incorporation into Brg1 chromatin remodeling complexes during embryo myogenesis. *Development* 141, 3378-3387. 10.1242/dev.108787 25078649 PMC4199139

[DEV159657C27] Gros J., Scaal M. and Marcelle C. (2004). A two-step mechanism for myotome formation in chick. *Dev. Cell* 6, 875-882. 10.1016/j.devcel.2004.05.006 15177035

[DEV159657C28] Gustafsson M. K., Pan H., Pinney D. F., Liu Y., Lewandowski A., Epstein D. J. and Emerson C. P. Jr. (2002). Myf5 is a direct target of long-range Shh signaling and Gli regulation for muscle specification. *Genes Dev.* 16, 114-126. 10.1101/gad.940702 11782449 PMC155306

[DEV159657C29] Hamburger V. and Hamilton H. L. (1951). A series of normal stages in the development of the chick embryo. *J. Morphol.* 88, 49-92. 10.1002/jmor.1050880104 24539719

[DEV159657C30] Horak M., Novak J. and Bienertova-Vasku J. (2016). Muscle-specific microRNAs in skeletal muscle development. *Dev. Biol.* 410, 1-13. 10.1016/j.ydbio.2015.12.013 26708096

[DEV159657C31] Hornstein E. and Shomron N. (2006). Canalization of development by microRNAs. *Nat. Genet.* 38, S20-S24. 10.1038/ng1803 16736020

[DEV159657C32] Ingham P. W. and McMahon A. P. (2001). Hedgehog signaling in animal development: paradigms and principles. *Genes Dev.* 15, 3059-3087. 10.1101/gad.938601 11731473

[DEV159657C33] Johnson R. L., Laufer E., Riddle R. D. and Tabin C. (1994). Ectopic expression of Sonic hedgehog alters dorsal-ventral patterning of somites. *Cell* 79, 1165-1173. 10.1016/0092-8674(94)90008-6 8001152

[DEV159657C34] Kahane N., Cinnamon Y., Bachelet I. and Kalcheim C. (2001). The third wave of myotome colonization by mitotically competent progenitors: regulating the balance between differentiation and proliferation during muscle development. *Development* 128, 2187-2198. 11493539 10.1242/dev.128.12.2187

[DEV159657C35] Kahane N., Ribes V., Kicheva A., Briscoe J. and Kalcheim C. (2013). The transition from differentiation to growth during dermomyotome-derived myogenesis depends on temporally restricted hedgehog signaling. *Development* 140, 1740-1750. 10.1242/dev.092726 23533174 PMC3621491

[DEV159657C36] Liu N., Williams A. H., Kim Y., McAnally J., Bezprozvannaya S., Sutherland L. B., Richardson J. A., Bassel-Duby R. and Olson E. N. (2007). An intragenic MEF2-dependent enhancer directs muscle-specific expression of microRNAs 1 and 133. *Proc. Natl. Acad. Sci. USA* 104, 20844-20849. 10.1073/pnas.0710558105 18093911 PMC2409229

[DEV159657C37] Liu N., Bezprozvannaya S., Shelton J. M., Frisard M. I., Hulver M. W., McMillan R. P., Wu Y., Voelker K. A., Grange R. W., Richardson J. A. et al. (2011). Mice lacking microRNA 133a develop dynamin 2-dependent centronuclear myopathy. *J. Clin. Invest.* 121, 3258-3268. 10.1172/JCI46267 21737882 PMC3148737

[DEV159657C38] Livak K. J. and Schmittgen T. D. (2001). Analysis of relative gene expression data using real-time quantitative PCR and the 2-ΔΔCT method. *Methods* 25, 402-408. 10.1006/meth.2001.1262 11846609

[DEV159657C39] Lopez-Rios J., Speziale D., Robay D., Scotti M., Osterwalder M., Nusspaumer G., Galli A., Holländer G. A., Kmita M. and Zeller R. (2012). GLI3 constrains digit number by controlling both progenitor proliferation and BMP-dependent exit to chondrogenesis. *Dev. Cell* 22, 837-848. 10.1016/j.devcel.2012.01.006 22465667 PMC4486391

[DEV159657C40] McDermott A., Gustafsson M., Elsam T., Hui C. C., Emerson C. P. Jr. and Borycki A. G. (2005). Gli2 and Gli3 have redundant and context-dependent function in skeletal muscle formation. *Development* 132, 345-357. 10.1242/dev.01537 15604102

[DEV159657C41] Mishima Y., Abreu-Goodger C., Staton A. A., Stahlhut C., Shou C., Cheng C., Gerstein M., Enright A. J. and Giraldez A. J. (2009). Zebrafish miR-1 and miR-133 shape muscle gene expression and regulate sarcomeric actin organization. *Genes Dev.* 23, 619-632. 10.1101/gad.1760209 19240126 PMC2658521

[DEV159657C42] Mok G. F. and Sweetman D. (2011). Many routes to the same destination: lessons from skeletal muscle development. *Reproduction* 141, 301-312. 10.1530/REP-10-0394 21183656

[DEV159657C43] Mok G. F., Mohammed R. H. and Sweetman D. (2015). Expression of myogenic regulatory factors in chicken embryos during somite and limb development. *J. Anat.* 227, 352-360. 10.1111/joa.12340 26183709 PMC4560569

[DEV159657C44] Mok G. F., Lozano-Velasco E. and Münsterberg A. (2017). microRNAs in skeletal muscle development. *Semin. Cell Dev. Biol.* 72, 67-76. 10.1016/j.semcdb.2017.10.032 29102719

[DEV159657C45] Münsterberg A. E., Kitajewski J., Bumcrot D. A., McMahon A. P. and Lassar A. B. (1995). Combinatorial signaling by Sonic hedgehog and Wnt family members induces myogenic bHLH gene expression in the somite. *Genes Dev.* 9, 2911-2922. 10.1101/gad.9.23.2911 7498788

[DEV159657C46] Murtaugh L. C., Chyung J. H. and Lassar A. B. (1999). Sonic hedgehog promotes somitic chondrogenesis by altering the cellular response to BMP signaling. *Genes Dev.* 13, 225-237. 10.1101/gad.13.2.225 9925646 PMC316396

[DEV159657C47] Pearse R. V. II, Vogan K. J. and Tabin C. J. (2001). Ptc1 and Ptc2 transcripts provide distinct readouts of Hedgehog signaling activity during chick embryogenesis. *Dev. Biol.* 239, 15-29. 10.1006/dbio.2001.0430 11784016

[DEV159657C48] Rao P. K., Kumar R. M., Farkhondeh M., Baskerville S. and Lodish H. F. (2006). Myogenic factors that regulate expression of muscle-specific microRNAs. *Proc. Natl. Acad. Sci. USA* 103, 8721-8726. 10.1073/pnas.0602831103 16731620 PMC1482645

[DEV159657C49] Rios A. C., Serralbo O., Salgado D. and Marcelle C. (2011). Neural crest regulates myogenesis through the transient activation of NOTCH. *Nature* 473, 532-535. 10.1038/nature09970 21572437

[DEV159657C50] Rosenberg M. I., Georges S. A., Asawachaicharn A., Analau E. and Tapscott S. J. (2006). MyoD inhibits Fstl1 and Utrn expression by inducing transcription of miR-206. *J. Cell Biol.* 175, 77-85. 10.1083/jcb.200603039 17030984 PMC2064500

[DEV159657C51] Sieiro D., Rios A. C., Hirst C. E. and Marcelle C. (2016). Cytoplasmic NOTCH and membrane-derived beta-catenin link cell fate choice to epithelial-mesenchymal transition during myogenesis. *Elife* 5, e14847 10.7554/eLife.14847 27218451 PMC4917337

[DEV159657C52] Sinha S. and Chen J. K. (2006). Purmorphamine activates the Hedgehog pathway by targeting Smoothened. *Nat. Chem. Biol.* 2, 29-30. 10.1038/nchembio753 16408088

[DEV159657C53] Smith T. G., Sweetman D., Patterson M., Keyse S. M. and Münsterberg A. (2005). Feedback interactions between MKP3 and ERK MAP kinase control scleraxis expression and the specification of rib progenitors in the developing chick somite. *Development* 132, 1305-1314. 10.1242/dev.01699 15716340

[DEV159657C54] Sweetman D., Goljanek K., Rathjen T., Oustanina S., Braun T., Dalmay T. and Münsterberg A. (2008). Specific requirements of MRFs for the expression of muscle specific microRNAs, miR-1, miR-206 and miR-133. *Dev. Biol.* 321, 491-499. 10.1016/j.ydbio.2008.06.019 18619954

[DEV159657C55] Teboul L., Hadchouel J., Daubas P., Summerbell D., Buckingham M. and Rigby P. W. (2002). The early epaxial enhancer is essential for the initial expression of the skeletal muscle determination gene Myf5 but not for subsequent, multiple phases of somitic myogenesis. *Development* 129, 4571-4580. 12223413 10.1242/dev.129.19.4571

[DEV159657C56] Trajkovski M., Ahmed K., Esau C. C. and Stoffel M. (2012). MyomiR-133 regulates brown fat differentiation through Prdm16. *Nat. Cell Biol.* 14, 1330-1335. 10.1038/ncb2612 23143398

[DEV159657C57] Ulloa F., Itasaki N. and Briscoe J. (2007). Inhibitory Gli3 activity negatively regulates Wnt/beta-catenin signaling. *Curr. Biol.* 17, 545-550. 10.1016/j.cub.2007.01.062 17331723

[DEV159657C58] Wen X., Lai C. K., Evangelista M., Hongo J.-A., de Sauvage F. J. and Scales S. J. (2010). Kinetics of hedgehog-dependent full-length Gli3 accumulation in primary cilia and subsequent degradation. *Mol. Cell. Biol.* 30, 1910-1922. 10.1128/MCB.01089-09 20154143 PMC2849461

[DEV159657C59] Wystub K., Besser J., Bachmann A., Boettger T. and Braun T. (2013). miR-1/133a clusters cooperatively specify the cardiomyogenic lineage by adjustment of myocardin levels during embryonic heart development. *PLoS Genet.* 9, e1003793 10.1371/journal.pgen.1003793 24068960 PMC3777988

[DEV159657C60] Yao C., Sun M., Yuan Q., Niu M., Chen Z., Hou J., Wang H., Wen L., Liu Y., Li Z. et al. (2016). MiRNA-133b promotes the proliferation of human Sertoli cells through targeting GLI3. *Oncotarget* 7, 2201-2219. 10.18632/oncotarget.6876 26755652 PMC4823029

[DEV159657C61] Yates A., Akanni W., Amode M. R., Barrell D., Billis K., Carvalho-Silva D., Cummins C., Clapham P., Fitzgerald S., Gil L. et al. (2016). Ensembl 2016. *Nucleic Acids Res.* 44, D710-D716. 10.1093/nar/gkv1157 26687719 PMC4702834

[DEV159657C62] Zeng L., Kempf H., Murtaugh L. C., Sato M. E. and Lassar A. B. (2002). Shh establishes an Nkx3.2/Sox9 autoregulatory loop that is maintained by BMP signals to induce somitic chondrogenesis. *Genes Dev.* 16, 1990-2005. 10.1101/gad.1008002 12154128 PMC186419

