# Supplementary material for: miR-133-mediated regulation of the Hedgehog pathway orchestrates embryo myogenesis
Source: Development. 2018 Jun 11;145(12):dev159657. doi: 10.1242/dev.159657 (PMC6031409; doi:10.1242/dev.159657)
Supplement: Supplementary information [file develop-145-159657-s1.pdf]

Supplemental figures

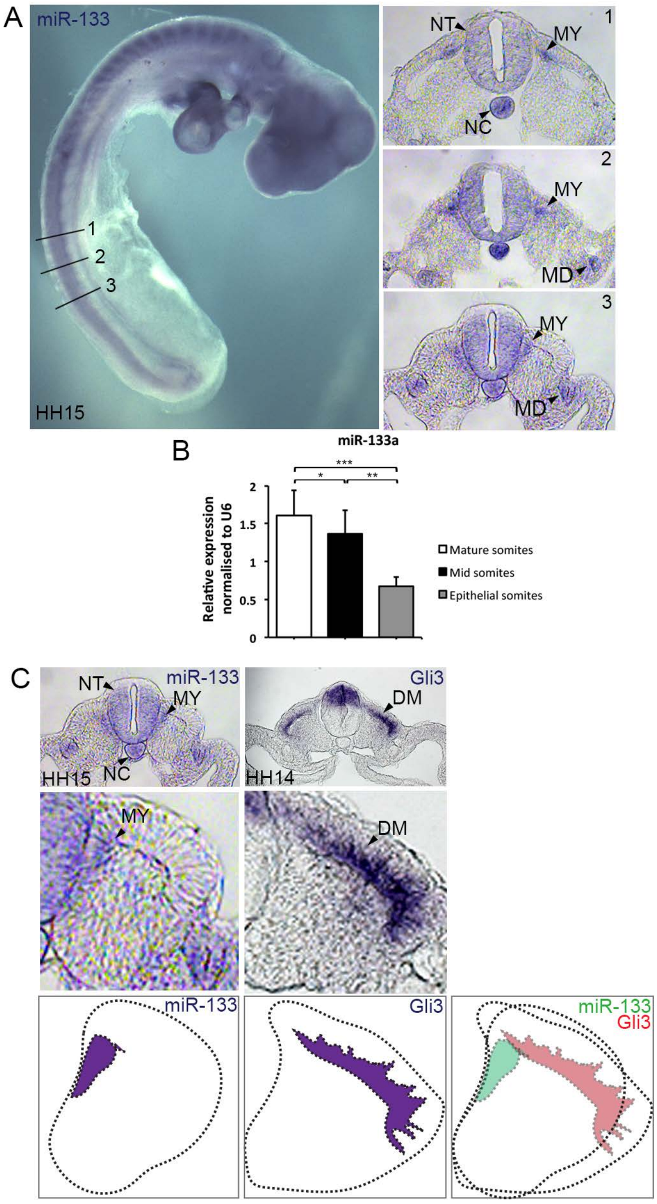

Fig. S1. Expression of miR-133 in developing somites. Whole-mount *in situ* hybridization using double digoxigenin-labelled LNA probes (Exiqon) shows restricted expression of miR-133 in the neural tube (NT), anterior notochord (NC) and medial somites, but no expression in the sclerotome (SC). (A) HH15 stage embryo. 1-3 indicates levels of cryosections shown in panels 1-3, with a schematic representing somite expression. (B) RT-qPCR for miR-133 shows a relative increase of expression in more mature, anterior somites compared with posterior epithelial somites. Eight biological replicates. ***P<0.001; **P=0.001-0.01; *P=0.01-0.1. (C) Comparison of miR-133 and Gli3 expression in developing somites. Higher magnification of somites show miR-133 expressed in nascent myoblasts (MY) and Gli3 in the dermomyotome (DM). MD, mesonephric duct.

Development • Supplementary information

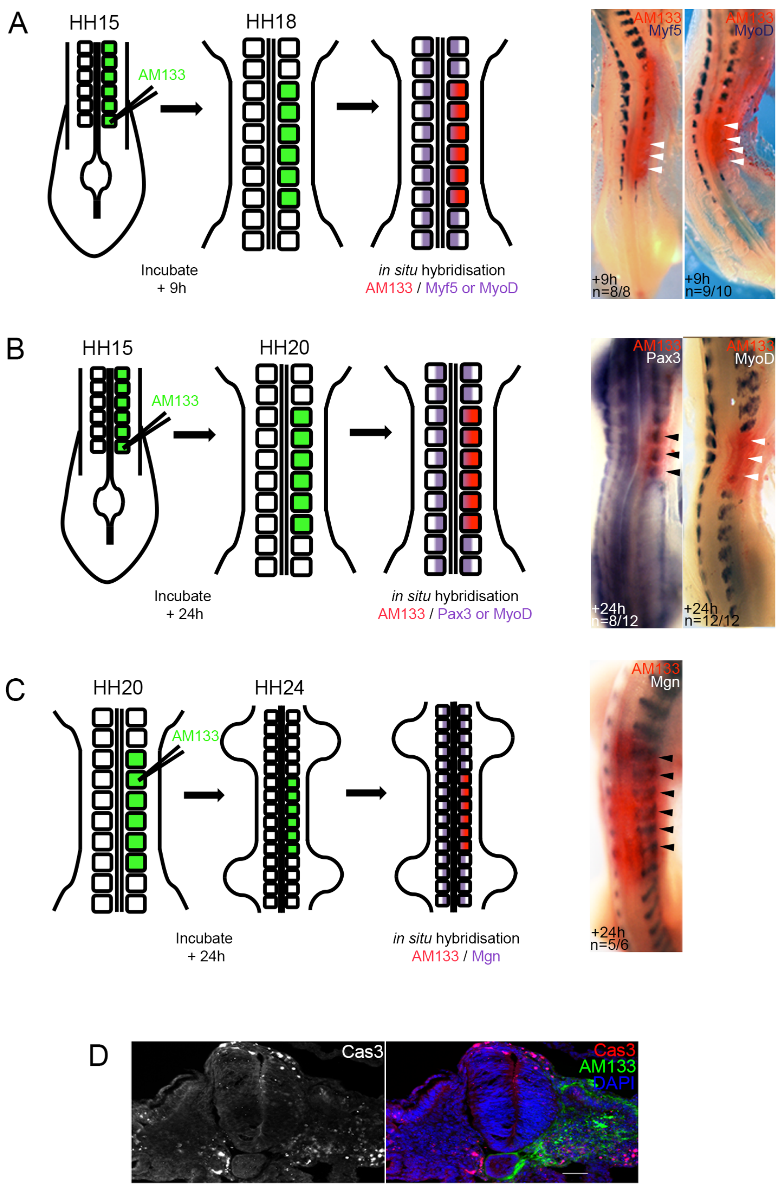

Fig. S2. Inhibition of miR-133 leads to a myogenic phenotype in early somites, but not in differentiating somites. Embryos were injected with FITC-labelled antagomir-133 (AM133) at HH14/15 or HH20. Whole mount *in situ* hybridisation was performed for Myf5, MyoD, Mgn or Pax3 as indicated. (A) 9 hour incubation, (B) 24 hour incubation post-injection. (C) AM133-injected into inter-limb somites at HH20 and incubated for 24 hours. Antisense probes (purple), FITC-labelled antagomir (red). Number of embryos indicated on each panel. White arrowheads indicating loss of expression on injected side; black arrowheads indicating reduced expression or no change in expression on injected side. (D) Immunostaining for Cas3, an apoptosis marker, in embryos injected with AM133 at HH14/15 and incubated for 9 hours show no difference in AM133 injected side compared to contralateral non-injected side. Scale bar 50 µm.

Development • Supplementary information

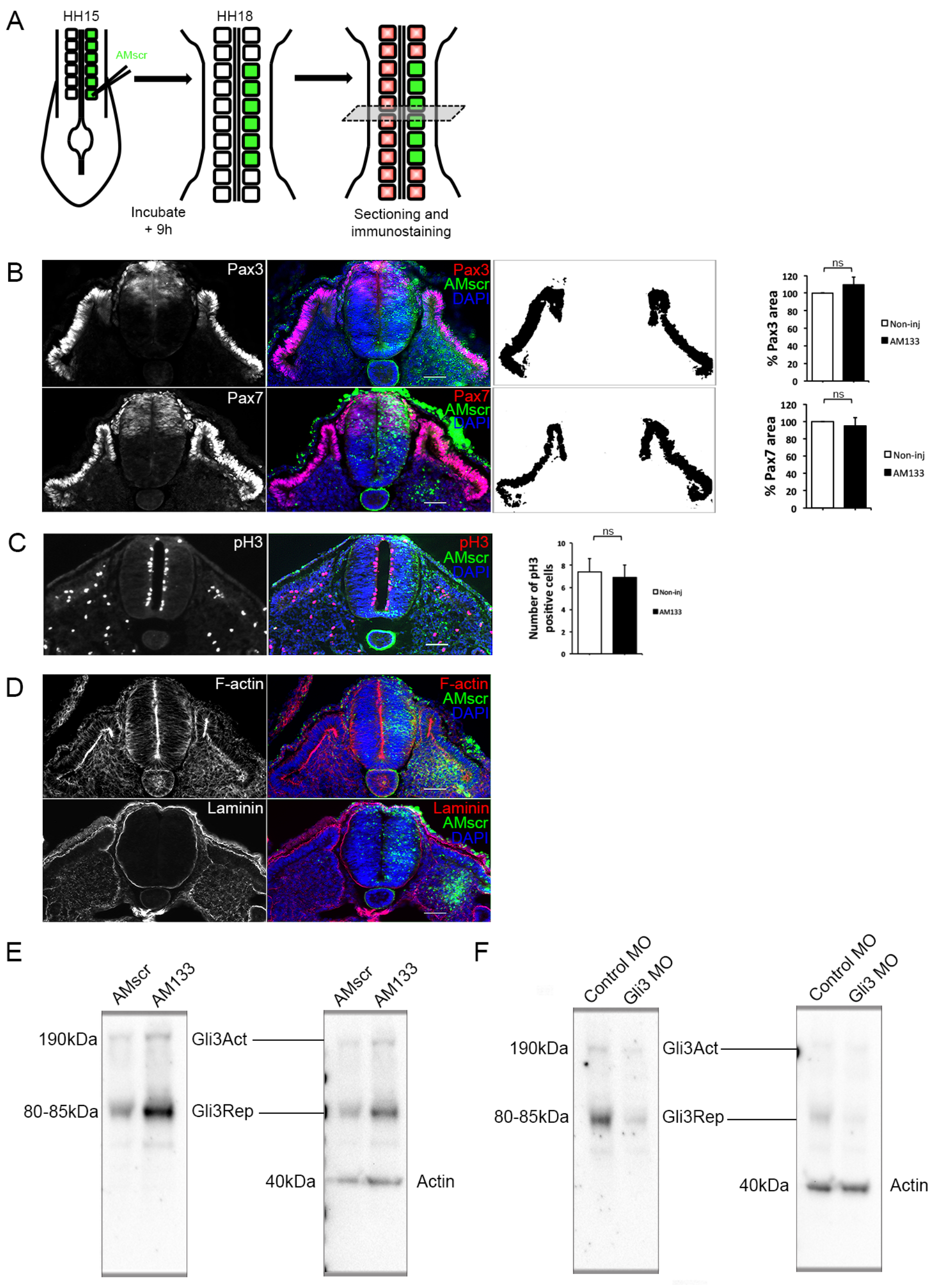

Fig. S3. Dermomyotome growth, epithelial organization and basement membrane deposition are not affected by injection of a scrambled antagomir. (A) Schematic representation of the experimental procedure. Somites of HH14/15 embryos were injected with FITC-labelled scrambled antagomir (AMscr) and processed for immunostaining after 9 hours. (B) Somites were cryosectioned and immunostained for Pax3, Pax7, FITC and DAPI as indicated. The areas positive for Pax3 or Pax7 staining within the somite were quantified using Fiji/ImageJ. There was no significant difference between injected and non-injected contralateral control side. (C) Immunostaining for pH3, FITC and DAPI as indicated. The number of pH3-positive cells was similar in AMscr injected somites compared to the contralateral side. Scale bar 50 µm; ns, not significant. (D) Immunostaining for F-actin, laminin, FITC and DAPI as indicated. Epithelial organization and basement membrane deposition were not affected and were similar on both sides. Scale bar 50 µm; ns, not significant. (E) Western blot detecting Gli3 protein in somites injected with AMscr or AM133 shows increased level of Gli3Rep

Development • Supplementary information

protein after miR-133 KD. (F) Western blot detecting Gli3 protein in primary cultured somites transfected with control MO or Gli3 MO shows reduced Gli3 protein after Gli3 MO.

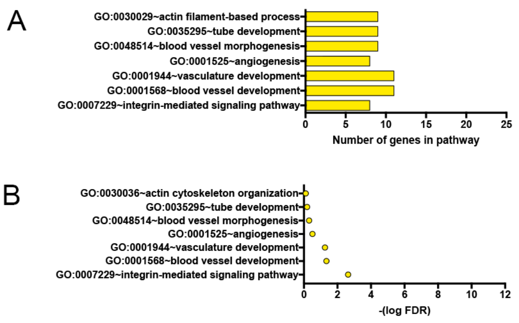

Fig. S4: Differential transcriptomics of somites after miR-133 KD. (A) Gene Ontology (GO) terms of genes expressed at higher levels in AM133 injected somites were associated with angiogenesis and tube morphogenesis. (B) However, the false discovery rates (FDRs) for these were significantly higher compared to GO terms associated with the downregulated DE genes.

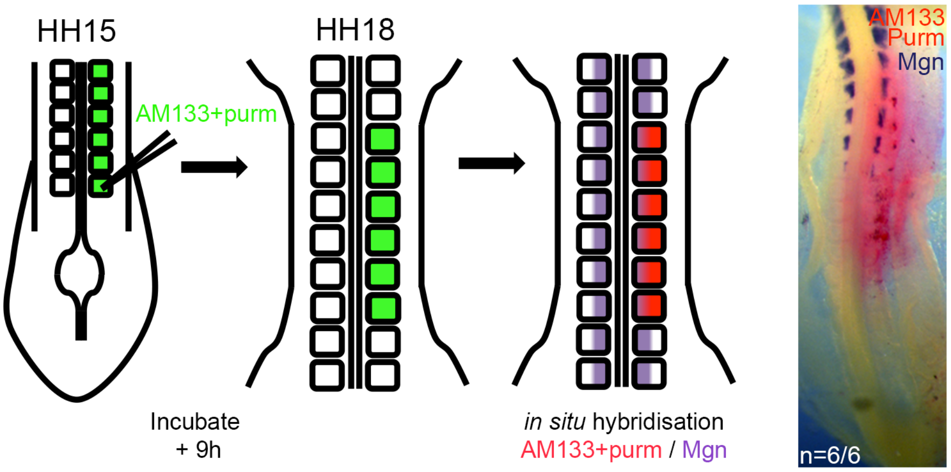

Fig. S5. Purmorphamine mediated activation of the Shh pathway rescues myogenic differentiation in absence of miR-133 function. Co-injection of FITC-labelled antagomir-133 (AM133) and purmorphamine (purm) into HH14/15 embryos was examined by *in situ* hybridisation for myogenin (Mgn) transcripts, which indicates normal expression after 9 hours incubation (n=6/6). Mgn antisense probe (purple), FITC-labelled antagomir (red).

Development • Supplementary information

Table S1 – PCR and mutagenesis primers, mimics and siRNA

cGli1 RT F	ACCTCTCCATCAGCACCATC
cGli1 RT R	TTGGCTGTCAGAGGGCTACT
cGli2 RT F	CACCATCAGCTCTGCCTACA
cGli2 RT R	TGAGCTGGTGTGAGGTTCAG
cGli3 RT F	CTCACCTCTTTCCAGCGTTC
cGli3 RT R	AATGTACGGGTGAGGAGTGC
cPtch1 RT F	GTGGAAGTTGGTGGACGAGT
cPtch1 RT R	CATGTACTCTGCTGGCCTGA
cPtch2 RT F	GAGTGAGGAGAAGGCAGGTG
cPtch2 RT R	CATGGTGACACAGGCATAGG
cSmo RT F	GACAACCCCAAGAGCTGGTA
cSmo RT R	CACAAAGAAGCAGGCATTGA
cHHIP RT F	CCCTCGACGATATGGAAGAA
cHHIP RT R	GTGGTGATCCACAGCACATC
cPax3 RT F	AGCAGAGCAACTGGAAGAGC
cPax3 RT R	GGTGGTTGAAAGCCATCAGT
cPax7 RT F	GCATCAAATTCGGGAAGAAA
cPax7 RT R	CTCTTCAAAGGCAGGTCTGG
cMyf5 RT F	CAACCCCAACCAGAGACTCC
cMyf5 RT R	GAGTCCGCCATCACATCGGA
cMgn RT F	GGCTTTGGAGGAGAAGGACT
cMgn RT R	CAGAGTGCTGCGTTTCAGAG
cCDC20 RT F	CCCTCAGCTGGAACAGCTAC
cCDC20 RT R	TGGTGCTGAGTGAAGGTCTG
cCDK1 RT F	TATAAAGGGCGCCACAAAAC
cCDK1 RT R	TCTTGAGGTCCATGGAAAGG
cFGF8 RT F	AGCAGAGCCTGGTGACAGAT
cFGF8 RT R	TTTCCCCTTCTTGTTCATGC
cSNAI1 RT F	CGATGCTCAGACCAGGAAAT
cSNAI1 RT R	AAGGGCTTTTCACCAGTGTG
cGREM1 RT F	AGGCACTGCACATCACTGAG
cGREM1 RT R	TCAGGGCAGTTGAGGGTAAC
cGAPDH RT F	TCTCTGGCAAAGTCCAAGTG
cGAPDH RT R	TCACAAGTTTCCCGTTCTCAG
cGLI3-UTR F	GCAGATCT TTTCACTTTCCACAAATCTGG
cGLI3-UTR R	ATGCTAGC TCAATCTGCC CAGTGAAATT A
cGLI3-UTR-MUT F	GCCTTATTTTGTGTTATCCTTTAACAGAGAGTTGACAAGATACGTTGACAAAT TTTCACAATGAGG
cGLI3-UTR-MUT-R	CCTCATTGTGAAAATTTGTCAACGTATCTTGTCAACTCTCTGTTAAAGGATAA CACAAAATAAGGC
c-miR-133a mimics	5'-UUGGUCCCCUUCAACCAGCUGU-3'<br>5'-AGCUGGUAAAAUGGAACCAAA U-3'
Sigma control siRNA	MISSION siRNA Universal negative control (#1) Sigma; product number SIC001
Gli3 MO	5'- ACGTATCTTGGTCCCTCTCTGTTAA-3'
Control MO	Standard control from GeneTools

Development • Supplementary information
